# Importance of sleep for avian vocal communication

**DOI:** 10.1098/rsbl.2022.0223

**Published:** 2022-08-17

**Authors:** Juliane Gaviraghi Mussoi, Margaret C. Stanley, Kristal E. Cain

**Affiliations:** School of Biological Sciences, The University of Auckland, Auckland, New Zealand

**Keywords:** sleep, birdsong, song learning, song performance, vocal communication, sleep deprivation

## Abstract

Sleep is one of the few truly ubiquitous animal behaviours, and though many animals spend enormous periods of time asleep, we have only begun to understand the consequences of sleep disturbances. In humans, sleep is crucial for effective communication. Birds are classic models for understanding the evolution and mechanisms of human language and speech. Bird vocalizations are remarkably diverse, critical, fitness-related behaviours, and the way sleep affects vocalizations is likely similarly varied. However, research on the effects of sleep disturbances on avian vocalizations is shockingly scarce. Consequently, there is a critical gap in our understanding of the extent to which sleep disturbances disrupt communication. Here, we argue that sleep disturbances are likely to affect all birds' vocal performance by interfering with motivation, memory consolidation and vocal maintenance. Further, we suggest that quality sleep is likely essential when learning new vocalizations and that sleep disturbances will have especially strong effects on learned vocalizations. Finally, we advocate for future research to address gaps in our understanding of how sleep influences vocal learning and performance in birds.

## 1. Introduction

Sleep-like behaviour is ubiquitous, found in every animal studied to date, from jellyfish and flatworms to birds and mammals [1–4]. Given that sleeping animals cannot perform fundamental activities (e.g. eating, reproducing, vigilance), sleep likely provides essential functions, and selection pressure must be high to maintain the behaviour [5,6]. Though the function of sleep is still unclear [1–3], we do know that sleep deprivation can have profound effects on many behaviours. In mammals, lack of sleep can affect the development of the brain, alertness, learning, memory consolidation and communication [7–11]. In birds, sleep disturbance has been shown to impair filial imprinting, cognitive performance and motivation [12,13].

The importance of sleep for vocal communication has primarily been demonstrated in humans. Sleep deprivation affects adults' speech performance components, such as word generation and intonation, impairing the ability to express thoughts and concepts [10]. Sleep also consolidates new language during learning, protecting it from subsequent interference and restoring decayed memory [14]. Children with more consolidated sleep during the first 2 years of their life have better language skills when they are 5 years old [15].

Vocal communication is essential in many animals' social interactions and is especially important in most birds, who use vocalizations for species recognition, mate attraction and resource defence (food sources, territories, mates) [16]. Most bird taxa use innate calls, but a few groups learn their vocalizations. Close-ended vocal learners (some oscine songbirds) learn only during a narrow window in development. However, other oscine songbirds, hummingbirds and parrots are open-ended learners, learning songs and calls throughout their lives [17]. Learning and maintaining vocalizations is a cognitive ability; it requires acquiring, processing and storing information [18]. Whether learned or not, many vocalizations require complex coordination of multiple systems (e.g. respiratory, muscle) [19].

Bird vocalizations are extraordinarily diverse, ranging from simple, monotone calls to incredibly complex mimicry of other birds, anthropogenic sounds or human voices [20]. Because vocalizations can be complex and coordinated behaviours, they are vulnerable to degradation, and poor vocal performance can have detrimental effects on fitness [19]. Research on daytime disturbances has shown that vocal learning and performance can be negatively affected by vegetation density [21], noise and light pollution [22,23], stress [24] and parasites [25], with associated negative effects on reproductive success and survival [16]. However, though sleep has strong effects on communication in other taxa, the effect of sleep, or the lack of it, on bird vocalizations is largely unknown.

Here, we argue that sleep disruptions likely have important and underappreciated consequences for bird vocal learning and performance. We highlight the extensive gaps in our current knowledge, make predictions about how bird taxa might be affected by sleep disturbances, and provide suggestions for future research in the field.

## 2. Bird vocalizations and sleep

### (a) Vocal performance and sleep

To emit even a simple vocalization, birds must coordinate their bill, syrinx, respiratory system and brain. Any interference with the regulation of these mechanisms can affect their ability to perform vocalizations [19]. Specific vocal features (e.g. duration, rate, frequency, amplitude) are crucial for species recognition and effective intraspecific interactions, as well as effective measures of individual quality or condition [18,20,26,27]. Some vocal features also depend on motivation; e.g. birds spend more energy and sing higher quality songs in more intense social interactions [28].

Sleep deprivation has vast consequences for animal physiology (altering body mass, temperature, hormone levels) and behaviour (hindering attentiveness, motivation, reaction times, coordination, emotional stability and increasing stress behaviour) [29]. Low motivation can hamper vocal output, altering vocal quantity, quality and timing [19,28]. Support for this possibility stems from recent research on the effects of artificial light on bird vocalization. Birds in areas with higher artificial light intensity tend to start singing earlier and stop singing later in the day [30,31]. In a laboratory setting, adult Japanese quails (*Coturnix japonica,* innate vocalizer) and adult zebra finches (*Taeniopygia guttata*, close-ended learners) in a constant light environment show decreased crow and song length [32]. Similar consequences are likely in all bird taxa, though to different degrees. These correlational studies are strongly suggestive, but to date, there has been only one study explicitly investigating the effects of direct sleep deprivation on bird vocal performance. Captive Australian magpies (*Cracticus tibicen,* open-ended song-learners) sing fewer but longer songs after sleep deprivation [13].

During sleep, old and new memories are enhanced, stabilized and replayed via memory consolidation and reconsolidation [33,34]. Many vocal learners crystallize some or all vocalizations by the time they reach sexual maturity, yet crystallized songs' stability depends on constant auditory, neurological and motor maintenance [17,35,36]. In adult zebra finches, the same neurons that activate when singing during the day are also activated during sleep, suggesting replay occurs [37–43]. Motor replay has also been observed via movement of the vocal organ muscles of zebra finches and some suboscine species (innate vocalizer) while asleep [43,44]. These pieces of evidence suggest that sleep plays a role in maintaining vocalizations, even when the vocalization is innate or already crystallized. If auditory, neurological and motor maintenance is disrupted due to poor sleep, vocal performance will likely degrade.

### (b) Vocal learning and sleep

Song learning consists of a developmental period followed by the crystallization of new songs [16]. Close-ended vocal learners only learn during a narrow developmental window, and those vocalizations are retained for life [45]. Open-ended vocal learners go through a similar vocal development phase but maintain varying degrees of plasticity, allowing them to continue learning new vocalizations or modify existing ones throughout their lives [45]. There is tremendous variation in the timing and length of learning stages; some close-ended learners crystalize within three months, while others take up to a year [46]. The ‘end goal’ of vocal learning also varies depending on the species. For some birds, having songs similar to their neighbouring conspecifics or tutors is advantageous, while for others, complex and unique songs are highly preferred for mate selection [47,48].

There is extensive evidence that sleep is critical for learning, memory and neuronal plasticity [49]. Nevertheless, little is known about how sleep affects vocal learning in birds. Both young zebra finches and varied tits (*Poecile varia*) learn songs during the day, and during sleep, these songs deteriorate [50,51]. Individual zebra finches that showed the greatest daily deterioration better matched their tutors after crystallization. In varied tits, sleep influenced their songs' maximum frequency. This indicates that deterioration during sleep is essential and may allow young birds to better match their tutors and prevent them from prematurely crystallizing inaccurate songs [34].

**Figure 1.**
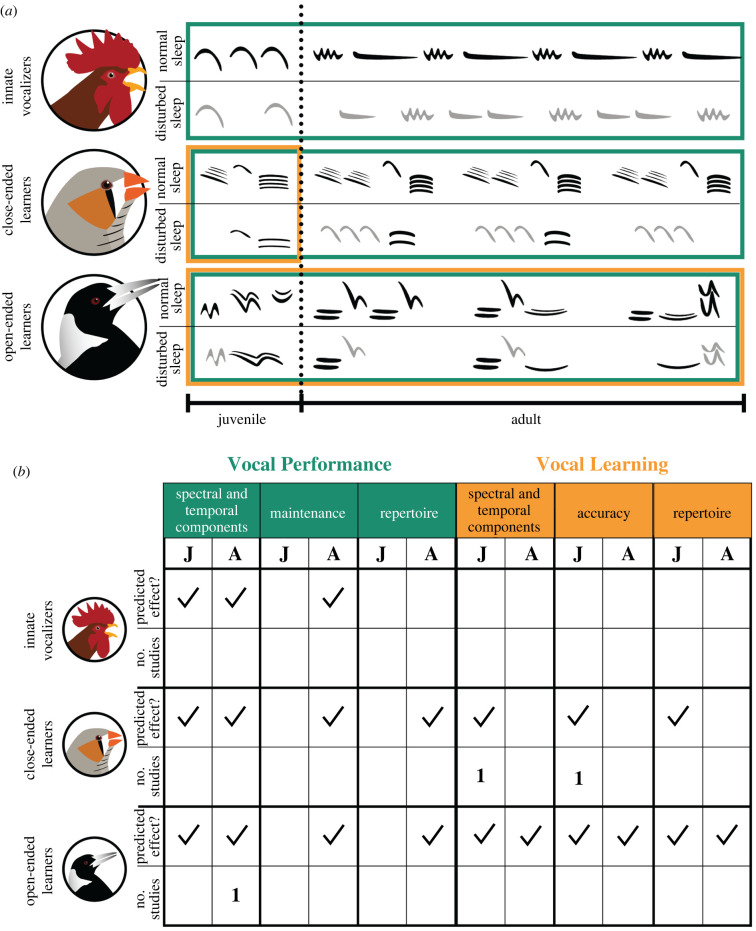
(*a*) Top rows are simplified vocalization spectrograms through life in different types of avian vocalizers, bottom row illustrates hypothetical alterations due to sleep disturbances. Note the missing elements, changed number and length of syllables and lighter colour—indicating lower amplitude. Coloured outlines indicate when vocal performance (green) or learning (yellow) are predicted to be affected by sleep disturbances. Dashed line indicates sexual maturity. (*b*) Table indicates vocal performance and vocal learning parameters predicted to be affected by sleep disturbance according to life stage (J, juvenile birds; A, adult birds). Spectral and temporal components include: amplitude, bandwidth, element length, output; maintenance = neuronal and motor maintenance of learned songs; repertoire = number of different songs or elements; accuracy = similarity to vocal tutor or mimicked sound. Numbers indicate the number of studies that explicitly test relationship between sleep (not only the lack of it) and these vocal parameters [13,50,51].

Open-ended birds continue learning and modifying songs throughout their lives; thus, sleep is likely to have similar effects on song learning in adulthood for these species (figure 1). There are no studies testing this possibility; however, adult starlings are better at discriminating between two similar songs after a sleeping period than after a comparable period of wakefulness [52,53], demonstrating the importance of sleep consolidation for learning auditory discrimination.

### (c) Predicting the effects of sleep disturbance on bird vocal performance

While there are good reasons to predict that sleep, and lack of it, are likely to influence birds' vocalizations, only a few species have been studied. This lack of broader research is problematic given that the extreme diversity of bird vocal learning and performance suggests there will be equally diverse responses to sleep disturbances. Here, we make some initial predictions about how different groups might respond to sleep disturbances (figure 1). We predict these effects are cumulative, i.e. effects on innate vocalizers will apply to close-ended learners, and effects on close-ended to open-ended learners. All effects will likely vary based on the degree and duration of sleep disturbance and individual characteristics (e.g. age, breeding stage, sex, condition).

#### (i) Innate vocalizers

We predict that lack of sleep would affect the vocal performance of both juvenile and adult innate vocalizers, such as chickens (*Gallus gallus domesticus*). By hindering the regulation of physiological mechanisms affecting motivation and stress levels, sleep disturbances might cause changes in the timing, quantity and quality of innate calls. For instance, disturbed sleepers may call less frequently, produce calls shorter in duration, or alter their temporal patterns. These changes are likely temporary, and innate calls should return to baseline levels after sleep recovery.

#### (ii) Close-ended vocal learners

Before crystallization, songs are moulded by the environment and vulnerable to anomalies via sleep disturbances. Because close-ended learners acquire their songs during a limited early development phase, we predict sleep disturbances during this phase to be particularly damaging to vocal learning processes. Elevated stress hormones hinder song learning; if sleep deprivation is stressful, this could indirectly impede learning [54–56]. Sleep disturbances during the learning period are likely to reduce song repertoire and increase inaccuracies by interfering with memory consolidation. Chronic sleep disturbances during song development may lead to permanent negative consequences. The maintenance of already crystallized songs in adulthood is also likely dependent on sleep, i.e. without adequate memory consolidation and reconsolidation due to sleep disturbances, learned songs will deteriorate, affecting their overall performance. In this case, we may expect shorter songs with fewer song elements, reduced stereotypy and poor structure or syntax.

#### (iii) Open-ended vocal learners

Open-ended learners retain a high level of vocal plasticity as adults and continue to modify current vocalizations and add new ones to their repertoire throughout their lives. Therefore, in addition to the critical nature of sleep during juvenile song development, we predict that for open-ended learners, sleep is essential for song learning throughout their adult lives. Sleep disturbances in adult open-ended learners may reduce the number of songs added to their repertoire, and learned songs may have fewer elements, reduced bandwidth, low amplitude, or poor fidelity. Some adult open-ended learners only learn new songs and modify old ones during specific periods of the year (e.g. canary—*Serinus canaria*) [57]; therefore, sleep disturbances are likely to be especially detrimental during those periods. Moreover, when new songs are crystalized, sleep disturbances may still affect song performance similarly to innate vocalizers and close-ended birds.

### (d) Future directions

Despite scarce empirical data, we argue that sleep disruption and disturbance likely have important consequences for avian vocal learning and performance. In addition to basic research testing for any such changes (figure 1), we suggest three key research directions for building an integrated understanding of the importance of sleep for avian vocal communication:

(i) It is imperative that future research include taxa with different vocal development patterns to fully understand the effects of sleep disturbances on bird vocalizations. Considering the current gaps, future studies should investigate the effects of sleep on the vocal communication of innate vocalizers, open-ended learners and adult birds.
(ii) Most bird sleep studies are conducted in a laboratory setting. Captivity can be stressful for birds and may cause abnormal behaviour and physiological responses [58,59]. Sleep patterns also vary depending on multiple extrinsic (e.g. season, presence of predators, parasites) and intrinsic factors (territoriality, foraging, reproductive activity and migration) [60]. In addition, some species might be adapted to the lack of sleep, compensate by sleeping deeper or for longer, and wild birds might avoid the source of disruption [61–63]. Hence, the effects of sleep on vocal learning and performance in wild birds are doubtlessly more complex than laboratory studies can reveal. Future research should also include less invasive methods to assess sleep in wild birds to increase accuracy, broaden taxa and minimize ethical issues [64].
(iii) With the continuous expansion of urban areas, anthropogenic nocturnal disruptions (e.g. light and noise pollution) are increasingly common and can interrupt birds' sleep patterns [23,65–67]. To date, only the direct effects of daytime noise pollution on vocalization have been studied, and generally from the perspective of signal transmission interruption from sender to receiver [68]. Light pollution studies have focused on circadian rhythms and daily activity changes [30]. However, the potential changes in vocal performance connected to light and noise pollution may be due to indirect effects mediated by the interruption of their sleeping patterns. By disentangling the direct and indirect effects of different sleep disturbances on bird vocalizations, we will be better able to predict and mitigate the consequences of urbanization on birds' behaviour.

Both vocal communication and sleep are part of complex systems, and their interactions are undoubtedly connected to multiple ecological and physiological factors. Therefore, we must start to explore these relationships and how different facets of vocal communication depend on sleep. Vocalizations are essential tools for most birds, crucial for attracting mates and defending territories. Vocal changes caused by sleep might have downstream consequences for reproductive success, survival and consequently, population dynamics. Finally, birdsong is a long-standing model for understanding human speech and language [18,69]. Thus, a clear understanding of the effects of sleep disturbance on learning and performance is likely to provide critical insight into human communication and sleep.

## Data Availability

This article has no additional data.
